# Heat stress and workload associated with sugarcane cutting - an excessively strenuous occupation!

**DOI:** 10.1186/2046-7648-4-S1-A23

**Published:** 2015-09-14

**Authors:** Rebekah AI Lucas, Theo Bodin, Ramon García-Trabanino, Catharina Wesseling, Jason Glaser, Ilana Weiss, Emmanuel Jarquin, Kristina Jakobsson, David H Wegman

**Affiliations:** 1 School of Sport, Exercise and Rehabilitation Sciences, University of Birmingham, UK; 2 Unit of Occupational Medicine, Karolinska Institutet, Sweden; 3 Scientific Board, Department of Investigation, Hospital Nacional Rosales, El Salvador; 4 La Isla Foundation, IL, USA/El Salvador; 5 Agency for Agricultural Health and Development (AGDYSA), El Salvador; 6 Occupational and Environmental Medicine, University of Gothenburg, Sweden; 7 Department of Work Environment, University of Massachusetts Lowell, MA, USA

## Introduction

Chronic kidney disease not associated with traditional risk factors (sometimes called Mesoamerican nephropathy) is prevalent in male agricultural labourers, particularly sugarcane cutters, in Central America and Mexico regions [1]. Strenuous work in a hot environment with dehydration is believed to be a key causal factor [1]. The aim of this study was to assess the level of heat stress and workload in sugarcane cutters.

## Methods

45 sugarcane cutters (34(12) y; range 18 - 57 y) from El Salvador were studied during the 2015 harvest (Feb-April). Heart rate (HR, Polar) was recorded in 10-11 workers per day, during 7 workdays. Weather data was collected using two weather stations (Weatherhawk, QuesTemp °34). Outdoor Wet Bulb Globe Temperatures (WBGT) was calculated (WBGT (outdoor) = 0.7WB + 0.2G + 0.1DB) via the QuesTemp °34. HR data were expressed a percentage of maximal HR (%HR_max_). A regression equation was used to predict HR_max _(208 - 0.7 × age) [2].

## Results

Sugarcane cutters worked on average for 7:30 hours (range 3:20 - 9:36 hours). In the field, WBGT reached 32.1°C (95% confidence interval [CI]: 33.0°C to 31.1°C), with 79 % (95% CI: 87 to 71%) of the day spent working at a WBGT above 26°C (threshold limit for continuous harvesting at 100 % [3]). Heart rates averaged 54 %HR_max _(95% CI: 57 to 52 %HR_max_) across all workdays. Workers spent 4:44 hours (95% CI: 5:19 to 4:09 hours) working at ≥50%HR_max _and 2:48 hours (95% CI: 3:21 to 2:15 hours) working <50%HR_max_.

## Discussion

Sugarcane cutting is repetitive high-intensity work carried out in high heat stress conditions. Workers spent over half the workday (including rest breaks) working at and above 50% of their HR_max_. This HR intensity is similar to that exhibited in the first 12 hours of adventure racing (64%HR_max _[4]) and higher than that maintained by soldiers during multi-day operations (30 - 40% of aerobic power [5]).

## Conclusion

The cardiac strain of sugarcane cutting is similar to that associated with very prolonged, competitive exercise and higher than that typically associated with self-paced hard work. Yet, sugarcane cutters maintain this work intensity daily throughout the harvest (~6 months).

**Figure 1:**
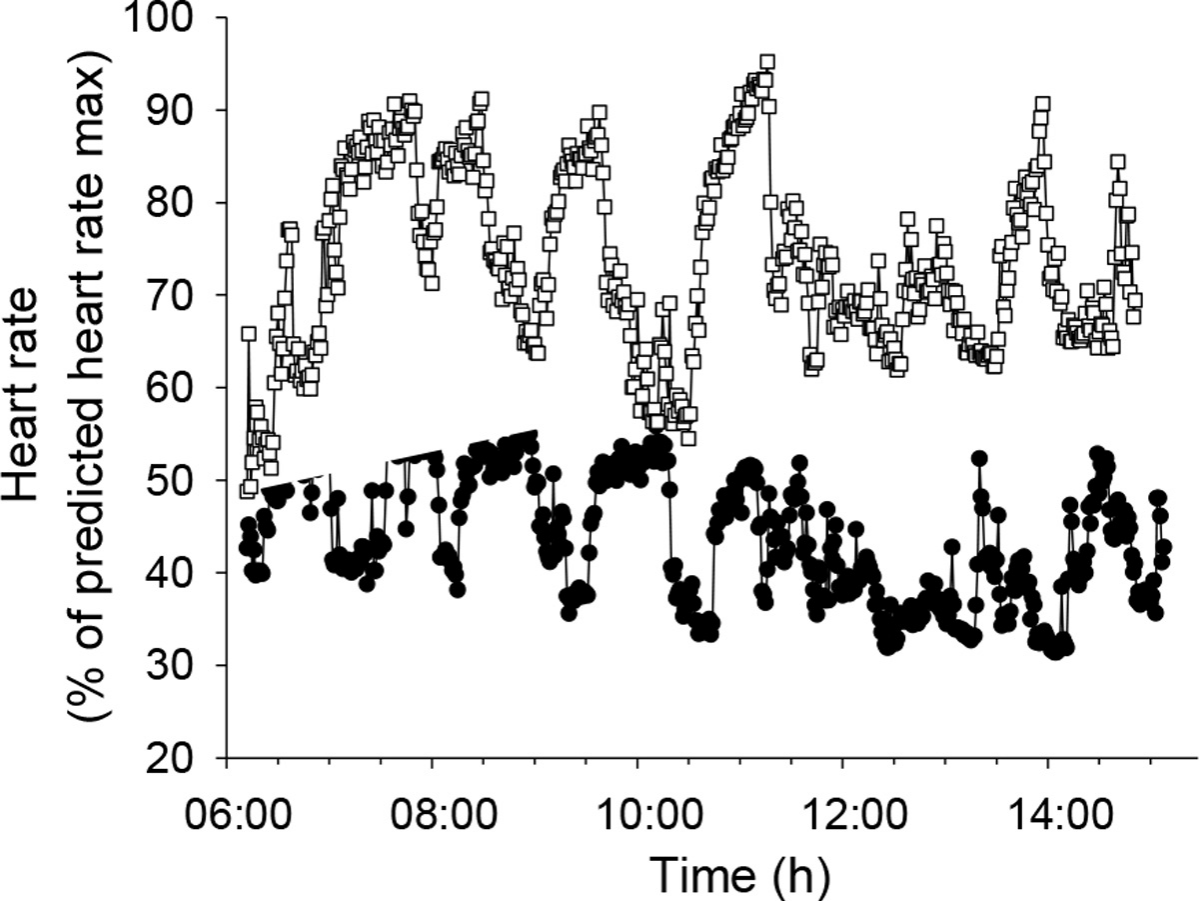
**Heart rate (1 min intervals) during one workday for two sugarcane workers**.
